# Blind Spots in Water Management, and How Natural Sciences Could Be Much More Relevant

**DOI:** 10.3389/fpls.2019.01742

**Published:** 2020-02-03

**Authors:** Ignacio Cazcarro, Jorge Bielsa

**Affiliations:** ^1^ Fundacion Agencia Aragonesa para la Investigacion y el Desarrollo (ARAID), Zaragoza, Spain; ^2^ Department of Economic Analysis, Faculty of Economics and Business Studies, Agrifood Institute of Aragon (IA2), Zaragoza, Spain

**Keywords:** plant water use and dynamics, water cycle and balance, water footprint, socioeconomic analysis, economic and political ecology

## Introduction

Estimates of crop evapotranspiration (ET) to measure the freshwater use indicator water footprint (WF) have undoubtedly been popular and implemented (Chapagain and Hoekstra, 2004), as well as the more recent extension to subnational regions and watersheds (Mekonnen and Hoekstra, 2010a; Hoekstra and Mekonnen, 2012; Mekonnen and Hoekstra, 2012). As reviewed by (Chenoweth et al., 2014; Lovarelli et al., 2016), these studies have gone from estimating products’ water trade on a global scale, to rigorous quantification for specific crops and geographical areas. Many studies have extended the coverage and precision of estimates. However, when it comes to the implementation of these improvements in local and river-basin water management, we find management problems that are ultimately unaddressed. It is here that, in our opinion, the Plant Water Sciences (PWS) have to shed light on these “blind spots.” We also illustrate these general ideas with two examples.

### Blind Spots in Recent Literature on Water Footprint: Fields for Future Research, Especially for Plant Water Sciences

Firstly, defining boundaries on what to account for human appropriation is a multidisciplinary and, to this point, open debate regarding WF calculations. Launiainen et al. (2014) questioned WF’s appropriateness for evaluating the water use in forestry and forest-based production. They pleaded for the exclusion of rain-fed forestry and forest-based production in WF, arguing that managed forest ET is indistinguishable from those of unmanaged forests. At a global level, there were case studies on some forest products such as paper WF (van Oel and Hoekstra, 2012), but these were not as systematic as those for crops and livestock WF. Nevertheless, we do need a clear split between both human and natural water usage in order to manage existing water resources.

Secondly, to further improve estimates, we find areas where WF can benefit from more precise studies of plant water ET and dynamics. Recent studies (Schyns et al., 2017; Schyns and Vanham, 2019) have estimated the WF (of production) of wood for lumber, pulp, paper, fuel, and firewood, but more can be done to calculate the WF on the consumer side, computing the responsibility of the demand (typically households/individuals) in the WF. Regarding livestock WF, the challenges involve discerning dry matter composition (concentrates/roughages). Furthermore, WF would probably benefit from updated estimates on roughages ET/WF, especially pasture/grass (estimated and briefly explained in Mekonnen and Hoekstra, 2010a). The relation and distinction of evaporation (E) to transpiration (T), absent in many studies until very recently, is very important from an economic perspective since T is *productive* and E is not (E can occur from soil but also from intercepted water on leaves). Recently, Nouri et al. (2019) found that mulching reduced irrigation needs by 3.6%, and when combined with drip irrigation, by 4.7%. There is thus an important need for studies on “partitioning” of ET and T (see review in Kool et al., 2014).

Thirdly, while there have been great advances in developing spatial and temporally explicit information, there is a divergence between the geographic and temporal units used by natural scientists and those used by social scientists for resource management (typically, river basins and long periods of time). If plant sciences do not provide information for the geographic areas in which decisions are taken, their work will be overlooked by social scientists. Regarding the temporal dimension, there are a lack of studies looking beyond a point in time (even when the evaluations of evapotranspiration are averaged over periods of time), and looking at the effects derived from land use and cover changes. In other words, studies looking at the dynamics of the resource rather than a static picture are needed. In summary, very detailed and methodical studies from the natural sciences coexist with rough approximations and simulations of data, such as those used by hydro-economic models (see for example Harou et al., 2009; Kahil et al., 2018 for a review of this form of work).

Fourthly, there are also blind spots regarding the estimation of historical WF time series. Historic economic analyses on water consumption usually rely on multiple sources of information: censuses, statistics on climate, precipitation, irrigation systems, agricultural production, yield, inputs used, water uses, etc. Information on crop water consumption (in m^3^ per unit of production) was also used in the past, relating it to scarcity and sustainability. How do we estimate the evolution of these coefficients over time? The answer likely lies in developing a methodology that allows us to obtain them from data on changes in irrigation systems, yields, harvest indexes, soils, etc. Dalin et al. (2012) and Duarte et al. (2014) initiated attempts to generalize coefficient changes over time based on yield changes. There is room for improvement in these estimates, e.g., incorporating not only the effect of changes in yield (as crop output per unit area), but also the changes in the harvest index (the ratio of grain yield to biomass when the crop matures), notably being increased (greater part of the biomass allocated to the grain) in many countries with the Green Revolution.

### Some Ideas on How to Improve Relevance in Practical Water Management

As happened previously with the concept of Integrated Water Resources Management (IWRM), a vast literature and discussion of a topic does not directly entail practical utility. One step further is needed. We cannot expect one indicator to be able to resolve everything, but we can provide additional data to complement it (e.g., Vanham et al., 2016, investigated whether the WF indicator addresses the food–energy–water ecosystem nexus, finding potential components to be included). Lund (2015) correctly highlighted that water management has always required not only physical sciences, but also social ones. Indeed, natural sciences have often not counted so much as it should on water management practices.

The present and future of the world’s food requirements and water needs have already been at the forefront since studies like those of Rockström et al. (1999). Greater water needs could lead to decisions that affect nature in the future. Derived from this, some researchers have tried to combine policy recommendations that take into account the management of local and global, acknowledging interrelations, especially between use and scarcity through trade (see Vörösmarty et al., 2015). However, the apparent remoteness of some phenomena (indirect chains of impacts) and the absence of monetary valuations beyond their relationship with agricultural production, have impeded their prominence in practice.

For the most part, economists’ contributions to the virtual water (VW) and WF literature have not been as comprehensive as it should either. These contributions have focused on: a) computing VW and WF through economic tools such as multi-regional input-output tables and models (Duarte and Yang, 2011; Tian et al., 2018); b) criticize or highlight limitations on the concept of VW based on the theory of comparative advantage (Wichelns, 2011; Gawel, 2014; Mateo-Sagasta et al., 2015; Wichelns, 2015); c) defend it or further explain factors completing the picture (Gawel, 2014; Afkhami et al., 2018; Zhao et al., 2019); and d) relate WF with scarcity and profitability, to obtain a visible ‘water productivity’ (Garrido et al., 2010; Cazcarro et al., 2019).

However, although most of these latest studies are focused on trying to reflect the shortage and opportunity costs, the recommendations of Lowe et al. (2018) for incorporating environmental valuation are necessary because it has not really been done. Along these lines, we extend this argument by stating that, in general, both economic valuations have not dealt with WF and economic analysis has not served to make WF socially relevant, which is a very desirable goal.

Therefore, we propose to go beyond the recommendations of Lowe et al. (2018), arguing that the economic valuation of water should also be based on measurements of WF, their scarcity, and the equivalence of their opportunity costs when regarding alternative uses or environmental costs generated.

There are other ways of making economic valuations of water, such as the literature of environmental valuation and that of ecosystem services (Martin-Ortega et al., 2015; Liu et al., 2016). The literature of hydro-economic models (HE) also pursues that goal (e.g., Escriva-Bou et al., 2018; Kahil et al., 2018).

We also emphasize the importance of working together with HE (more biophysical) models, and of incorporating broader perspectives and tools (political, economic, and social ecology). For example, Hellegers and van Halsema (2019) argue that valuation is very useful for decision-making and that it must go beyond economics. We also have examples of more social assessments (Rodríguez-Labajos and Martínez-Alier, 2015; Wright-Contreras, 2018) and more in the realm of ecological economics (Kallis et al., 2013; Gómez-Baggethun and Martín-López, 2015). All this literature has one element in common: it is about following, as Lund (2015) points out, a problem-based approach rather than a discipline-based approach.

If the above seems too “ethereal,” we propose a couple of concrete examples below.

### Two Examples for Practical Relevance

First example: the management of forests and other types of vegetation. This entails biodiversity, erosion, emissions, lack of management, which affects the risk of fires, but also its effect on water availability (in Blanco (2017), existing paradigms on the relation between forests and water are even challenged). Indeed, existing blue-green water relations and substitutability are often ignored by analyzing them as if they were two distinct areas. As reviewed in D’Odorico et al. (2018), decades of research on deforestation have highlighted the profound hydroclimatic impacts of land use and land cover change (Perugini et al., 2017). Although it is very difficult to generalize conclusions for different kinds of forests, climates, soils, etc. (for which more studies on grassland *vs*. agroforestry are needed), Kay et al. (2018) show consistently for six case studies in Europe how groundwater recharge rate tends to be higher in agricultural landscapes without agroforestry systems. On rain-fed farmland *vs*. forestry, the former is found to sustain lower evapotranspiration rates because of the smaller leaf area index, surface roughness, root depth, and greater albedo (Bonan, 2008; Perugini et al., 2017; D’Odorico et al., 2018). On closed *vs*. open forest, the first reduces more infiltration (Gracia et al., 2011; Di Prima et al., 2017). All in all, both ET and runoff go against infiltration. The reason is that, although truly runoff does not increase on site storing, water remains within the basin, while with ET water escapes from it. For example, in the Ebro basin, Bielsa et al. (2011) and López-Moreno et al. (2008; 2014) found surprisingly small water volumes in downstream gauges which lead them to identify increasing natural revegetation as a potential explanatory factor. We believe plant sciences have a great deal to contribute to river basin water management since land-use changes, particularly forest extent and coverage, can modify forest water demand and hence blue water availability. Some policy makers only take action when they grasp, if roughly, the economic implications of these water losses or water pollution.

Consider a second example. As highlighted by Shtull-Trauring et al. (2016), high-resolution studies can provide data to inform policy makers and farmers about the appropriate crops and cultivation practices that lower the WF by increasing water use efficiency and reducing the negative impact on the environment. On that basis, we would also add that policy makers will only take appropriate decisions knowing the whole picture of the benefits and costs for society. We know that the market economic valuation instruments are not usually appropriate for this cost-benefit calculations (see e.g., Van der Zaag and Savenije, 2006). Therefore, a key aspect is the consideration of many water services as public goods. This implies requiring some type of agency that considers social costs and benefits [be they public administrators/non-governmental organizations (NGOs), etc.] to manage it. Only if the water cycle (and thus the green side of it) is fully known, can informed and sound decisions be made. Clearly, plant water dynamics have much to contribute in this area. **Table 1** summarizes advances, gaps and relevance for PWS of a selection of the cited literature.

**Table 1:** Selected review of literature on water footprint (WF) notably related to plant water sciences (PWS) and dynamics, and key gaps for water management (WM).

Study/ies (physical)	Class and methods	Advances	Gaps or aspects not covered	Relevance for PWS and dynamics
(Mekonnen and Hoekstra, 2010a; Mekonnen and Hoekstra, 2010b; Hoekstra and Mekonnen, 2012; Mekonnen and Hoekstra, 2012)	ET, VW, and WF computation	WF of crop and livestock products.	Temporal dimension other than averages of a period (TDOTAOAP). Rough results on grasslands WFs.	Need of PWS to compute other temporal dimensions and grasslands WFs
(Liu et al., 2016) (Mekonnen & Hoekstra, 2010b)	VW and WF computation, notably of gray WF.	Gray WF computation making 1^st^ assumptions on leaching and run-off of nitrogen fertilizers.	TDOTAOAP. Lack of other pollutant than N. Lack of sensitivity analyses on key assumptions	Need of PWS to test sensitivity to some fixed assumptions on leaching and run-off.
(Schyns et al., 2015)	Review and classification of indicators of green water availability and scarcity.	Advances on defining and measuring green water scarcity.	Choosing and making operational the green water scarcity indicators discussed.	Need of PWS to determine which part of the green water flow is productive.
(Schyns et al., 2017; Schyns & Vanham, 2019)	ET, VW, and WF computation, notably of forestry products WF.	WF of forest products.	TDOTAOAP. Full accounting by more detailed tree types and locations.	Need of PWS to obtain results more detailed tree types and locations.
(Shtull-Trauring et al., 2016)	Combination of high resolution WFs with GIS to analyze impacts of different factors.	Finds insufficient classic WF yield parameter (m^3^/ton) alone to compare different crops; and strong impact in gray WF of the water quality standards used.	TDOTAOAP. Lack of wider picture of the full social benefit and costs.	An example to follow. Great attempt of linking and making useful PWS for WM at relevant scales.
(Nouri et al., 2019)	E and T distinction	E and T distinction, introducing the effect of mulching on WF.	Lack of study of the feasibility and practicality of the strategies proposed.	Shows the relevance of this distinction for WF studies (e.g., water-saving effect of mulching and drip irrigation).
**Study/ies (economics)**	**Class and methods**	**Advances**	**Gaps or aspects not covered**	**Relevance for PWS and dynamics**
(Wichelns, 2011; Mateo-Sagasta et al., 2015; Wichelns, 2015)	Critiques to WF related to comparative advantage	Some fair critiques to WF related to comparative advantage.	Full understanding of the searched goals with VW and WF, and the implications of comparative advantage	Show a wider picture of how PWS results feed and are fundamental for WF and derived global and comprehensive studies: water content of consumption, effects and scenarios from different diets, population projections, etc.
(Daniels et al., 2011; Duarte and Yang, 2011; Tian et al., 2018)	Multi-region models, input-output	Highlight the role of the full global chains for water (not just agri-food, energy, paper).	Typically, less precision in the agri-food WFs, and short temporal spans.	
(Garrido et al., 2010; Cazcarro et al., 2019)	WF, scarcity, and water apparent productivity	Highlight the role of dryland/irrigated land, and the relation of WF to scarcity and water apparent productivities.	Lack of other economic valuation of water and WF than that of the market (prices, monetary values, etc.).	Shows very different economic relevance of rainfed/irrigated/grass/land for WFs and the need of PWS to properly account them.
Dalin et al. (2012); Duarte et al. (2014), Konar et al. (2013)	Historical WFs computations	Attempts to get and use historical WFs to obtain relations of water pressures and socioeconomic variables	Simplistic methods of computing historical WFs based on yields	Researchers in PWS are better suited to obtain estimates of historical WFs.
(Lund, 2015; Lowe et al., 2018)	Review of integrating social with physical sciences for WM accomplishments. Comment on the need of linking WF and economic valuation. Literature review.	Highlight of the needs of linking natural and social sciences for water management, and of the use of economic valuation.	Focus only on a sub-discipline of environmental economics without citing ecological and political ecology options.	Shows the importance of problem-solving focus. Provides perspective on the valuation of ecosystem services, etc.
This Opinion article	Opinion on the cited WF blind spots and proposals to make more relevant PWS and economics in WM.	Identification of WF literature gaps especially related to PWS and proposals to make them more relevant in WM.	Lacks empirical application other than brief examples. Does not explain in full detail some of the arguments presented.	Finds blind spots in which PWS could shed light on WF calculation. Extends perspectives on how to make PWS research more useful and consistent with socioeconomic analysis for practical WM.

## Concluding Remarks

As we have argued based on the critical analysis of recent literature on water management and on WF, new advances in the study of plant water dynamics have great potential to improve the understanding and management of the water cycle. These have to do with the data from the ET measurements and their dynamics. This need is important for pastures, forests, and vegetation and, in particular, for economically and politically relevant areas and periods of time. The ultimate goal is to develop work based on problems and not on disciplines. However well-known that principle is, it is still not applied in many scientific areas.

## Author Contributions

IC and JB contributed equally to the analysis and writing of the article.

## Funding

IC acknowledges the financial support from the Ramón Areces Foundation, grant CISP15A3198.

## Conflict of Interest

The authors declare that the research was conducted in the absence of any commercial or financial relationships that could be construed as a potential conflict of interest.
